# A Phase Canceling Technique to Improve SAW Duplexer Isolation

**DOI:** 10.3390/mi14020239

**Published:** 2023-01-17

**Authors:** Jianbin Tao, Zhengjie Tang, Yali Zou, Bin Wang, Jiawei Li, Yuhao Liu, Tao Wu

**Affiliations:** 1 School of Microelectronics, Shanghai University, Shanghai 200444, China; 2 Changzhou Bowo Microelectronics Co., Ltd., Changzhou 213000, China; 3 School of Information Science and Technology, ShanghaiTech University, Shanghai 201210, China

**Keywords:** surface acoustic wave, duplexer, isolation

## Abstract

Spectrum resources are becoming increasingly crowded, and the isolation interval between different systems is getting smaller and smaller. This puts forward higher requirements for the duplexer. The duplexer is an important part of the radio frequency front end, and the isolation requirement is becoming higher. This paper presents a phase canceling circuit to improve the performance of the duplexer to meet the requirement of the communication system for isolation. A phase canceling circuit is an effective method to enhance the isolation through use of a surface acoustic wave (SAW) on-chip circuit. It contains a duplexer and a branch. The branch is designed for diminishing the leakage signal of the duplexer. Compared with the leakage signal, the branch consists of two attenuators and a phase shifter to generate a signal which has equal extent and reverse phase. As a result, this method is capable of increasing the isolation of band 5 by 12 dB in the downlink frequency. Meanwhile, it neither affects other factors, such as insertion loss or return loss, nor increases the size of the chip. The phase canceling circuit is expected to promote the quality of duplexer to satisfy the strict requirements in 4G and 5G systems.

## 1. Introduction

With the development of communication technology, multichannel concurrent communication requires a transmitter (Tx) and receiver (Rx) to use the same antenna (ANT) [1]. Therefore, the radio frequency front end must have synthesis and separation devices; that is, a duplexer. Scant frequency resources are becoming more and more precious. and interference between them is more serious, which puts forward higher requirements for the duplexer [2]. Because the frequency bands of the duplexer are adjacent to each other, and the harmonic will affect the quality of communication, the isolation between the two channels has become a key factor in the design of the duplexer [3].

In order to achieve higher isolation, in some existing technologies, a high Q value SAW resonator is used, such as temperature compensation surface acoustic wave (TC-SAW) and thin film surface acoustic wave (TF-SAW). They have a higher impedance ratio which can provide higher suppression [4]. However, the fabrication process is relatively complicated. Redesigning the package and bonding wire scheme also can improve the isolation. Adding ground vias between the package ground and inner ground plane can stop the electromagnetic coupling between the meander delay line and the other components [5]. As another existing method of improving the duplexer, isolation can also be implemented by improving the circuit structure. By using orthogonal linearly-polarized antennas and the transformer-based duplexer, the transmitter and receiver duplexes the two antennas with orthogonal circularly polarized signals [6]. Results show that the achieved isolation is better than 50 dB. Furthermore, magnetic tuning is introduced to improve isolation in cases of practical imbalances [7]. A feed-through model is added for adjusting measurements and simulations. A good agreement with this composite simulation and experiment can be obtained for this duplexer, with an isolation of 48 dB in the RX band. Moreover, there are some simple structures to improve the isolation, such as an external circuit and a matching component. These kinds of circuits often use an external signal to cancel out the leakage signal. The duplexer often has two kinds of leakage signals. One is the TX leakage via the signal line, and the other is caused by electromagnetic coupling and electrostatic coupling. Through use of a matching circuit at the antenna, the amplitude and the phase of the TX leakage can be adjusted to cancel out another leakage signal [8,9,10]. This duplexer has an isolation higher than 70 dB. In [11], an external circuit was added to cancel out the TX leakage. The circuit is designed by a capacitor, a transmission line, and an inductor to generate a feedback signal. In contrast with the TX leakage, this feedback signal has an equal amplitude and a reverse phase signal which can improve the isolation to 50 dB.

This paper investigates a SAW phase canceling circuit which can improve the isolation for the duplexer. The phase canceling circuit contains a duplexer that needs to be ameliorated and a branch to generate a feedback signal which is beneficial for the isolation. The branch has two interdigital capacitors to change the amplitude of the feedback signal and a double mode SAW (DMS) to transform the phase of the feedback signal. Important, the DMS is designed by phase-weighted interdigital transducers (IDTs) to control the phase more precisely. As a result, the isolation can be improved to 60 dB because the feedback signal cancels out the leakage signal of the duplexer.

## 2. Duplexer and Leakage Signal

There are many filter structures, such as the single-phase unidirectional transducer (SPUDT), transversely coupled resonator filter (TCRF), and DMS and impedance element filter (IEF). In recent years, the ladder-type filter and DMS have been commonly used. The ladder-type filter has good out-of-band rejection and insertion loss [12]. Another advantage of the ladder-type filter is that the power-handling ability is stronger than other structures [13]. Due to the good electrical properties and power-handling ability, this type is widely used in the TX filter and has an irreplaceable position. However, the disadvantage of the ladder-type filter is that its size is too large. If the TX filter and RX filter use ladder-type filters, the size of duplexer will be large. DMS can provide low insertion loss and high rejection, and it also does not need to use a combination of many resonators like the ladder-type filter which avoids occupying more die area [14]. However, it has a bad power-handing ability which means DMS can only be used at the RX port. The high-power signal in TX is bound to affect RX [15]. The structure of the ladder filter and DMS cannot meet the requirement of isolation.

Due to the limited attenuation, the signal in the TX filter will partly leak into the RX filter [16,17]. This leakage signal will have a serious impact on the performance of the duplexer [18]. In order to reduce this impact, it is important to improve the isolation of the duplexer.

**Figure 1:**
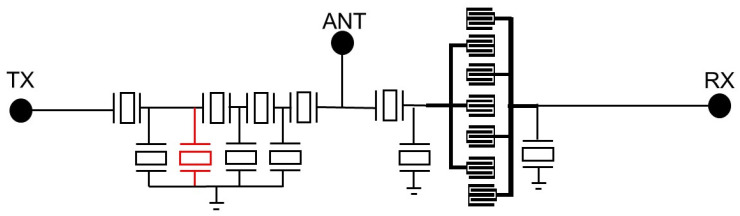
The structure of band 5. The red indicates the suppression resonator. TX—transmitter port; RX—receiver port; ANT— antenna port.

In this paper, a band 5 duplexer is discussed. As shown in Figure 1, the structure of the TX is a ladder-type filter. It has four series resonators and three parallel resonators. A suppression resonator is added in the parallel resonators which is parallel to the first parallel resonator. This resonator can generate a zero point to improve the isolation at 898 MHz. The RX is a composite structure which contains one series resonator, a seventh-order DMS, and two parallel resonators to ensure a broader bandwidth and a sharper filter skirt. 

**Figure 2:**
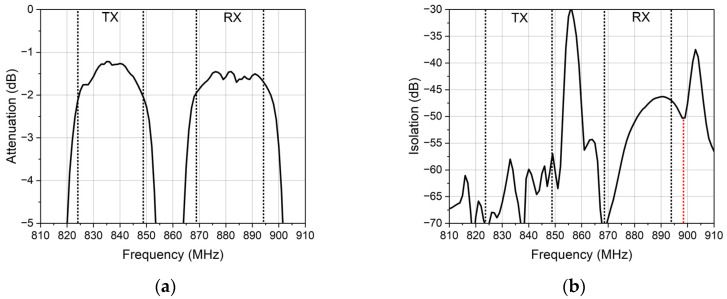
The simulation characteristics of the duplexer. (**a**) The insertion loss of the duplexer. (**b**) The isolation of the duplexer.

Figure 2 shows the simulation characteristics of the duplexer. Figure 2a shows that the insertion loss is lower than 2 dB. In order to obtain a favorable insertion loss, the isolation in the RX band is less than 50 dB, as shown in Figure 2b. Although the suppression resonator generates a zero point at 898 MHz. The zero point can improve the suppression performance outside the passband, but this isolation cannot meet the requirement. 

## 3. Phase Canceling Circuit

### 3.1. Principle of Phase Canceling Circuit

In order to reduce the leakage signal to improve the performance of the duplexer, the phase canceling circuit is designed to counteract the leakage signal to improve the isolation. The principle of the phase canceling circuit is as follows.

A feedback signal is added to reduce the leakage signal. Assume that the leakage signal is F and the feedback signal is $F^{\prime }$. To reduce the leakage signal, the feedback signal should meet the following requirements:

(1) $$\left|F^{\prime }\right|=\left|F\right|$$

(2) $$\left|∠F^{\prime }-∠F\right|=\pi$$

**Figure 3:**
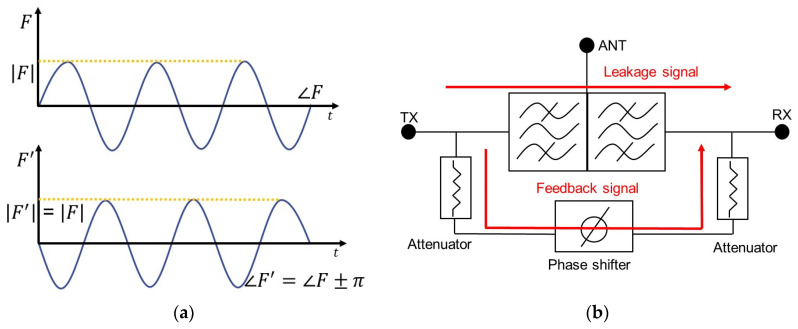
The principle of the phase canceling circuit. (**a**) The requirements of the feedback signal. The blue one is leakage signal and feedback signal. The yellow one is the amplitude. (**b**) The schematic diagram of phase canceling circuit.

As shown in Figure 3a, the amplitude of the feedback signal is the same as the amplitude of the leakage signal, and the phase of the feedback signal is the opposite to the phase of the leakage signal. Two signals with the same amplitude and the reverse phase can cancel out each other and the reduction of leakage signal can improve the isolation.

As shown in Figure 3b, a duplexer and a branch compose the phase canceling circuit. The branch contains two attenuators and a phase shifter to generate a feedback signal. Two attenuators control the amplitude of feedback signal and the phase shifter controls the phase of the feedback signal. Compared with the leakage signal, the feedback signal has equal extent and a reverse phase to cancel it out. In summary, the reduction of the leakage signal leads to a higher isolation.

### 3.2. Structure of the Phase Shifter

**Figure 4:**
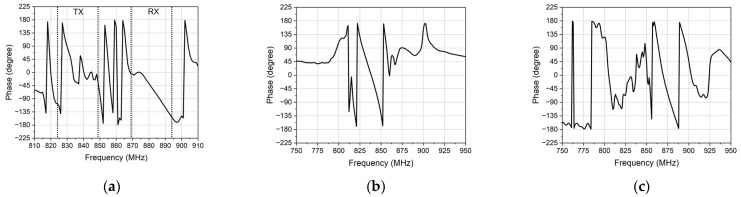
Different phases. (**a**) The phase of TX to RX. (**b**) The phase of the ladder type. (**c**) The phase of the DMS.

The software of this study was performed using Advanced Design System (ADS), which allows the phase to be observed by the ADS directly. Figure 4a shows the phase of TX to RX. The phase can change from 180 degrees to −45 degrees between 824 MHz to 849 MHz and from 0 degrees to −180 degrees between 869 MHz to 894 MHz. The goal of the phase shifter is to generate a reverse phase. Figure 4b,c show the phase of the ladder type and the phase of the DMS. The phase of ladder-type changes from 180 degrees to −180 degrees between 810 MHz to 850 MHz. The phase of the DMS changes from 180 degrees to −180 degrees between 780 MHz to 885 MHz. The variation range of DMS is larger than that of the ladder type and the size of the DMS is less than the size of the ladder type. This means DMS is suitable to be used as the phase shifter to reverse the phase through reasonable design [19,20]. In order to control the phase more precisely, the DMS is designed by phase-weighted IDTs.

**Figure 5:**
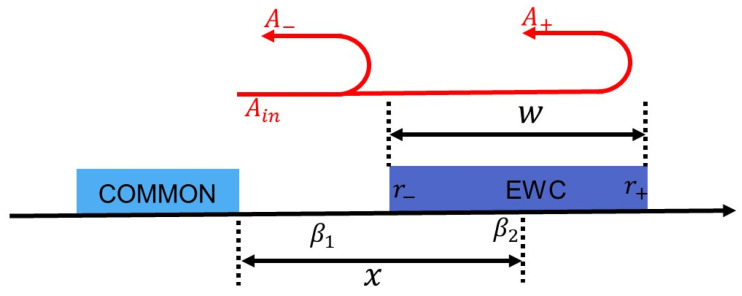
The reflection of the EWC electrode.

Electrode width-controlled (EWC) IDT is a kind of phase-weighted IDT [21,22,23,24]. The concept of EWC is that by controlling the width of interdigital electrode, the reflectivity will be changed to achieve the expected phase. Figure 5 shows the reflection of EWC electrode. The reflection is as follows:

(3) $$\;A_{-}=r_{-}A_{in}e^{-2j\beta_{1}\left(x-\frac{w}{2}\right)}$$

(4) $$A_{+}=r_{+}A_{in}e^{-j\left(2\beta_{1}x+\beta_{2}w\right)}$$

where $r_{-}$ and $r_{+}$ are the reflection coefficients at the left and right, $\beta_{1}$ is the wave number between the electrodes, $\beta_{2}$ is the wavenumber of the electrodes, x is the position of the center of the EWC electrode, and w is the width of the EWC electrode. The phase is related to β, x, and w. β is difficult to control, but x can be controlled by the number of EWC electrodes, and w can be controlled by the width of the EWC electrodes. The phase can be changed with the alteration of number and width of the EWC electrodes.

**Figure 6:**
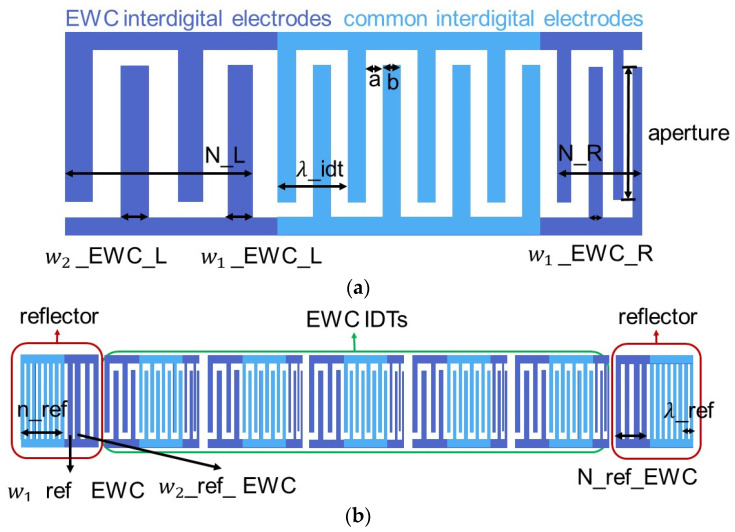
The structure of the phase shifter. (**a**) The structure of the EWC IDT. It contains the aperture, the number of the common interdigital electrode n_idt, the period of IDT λ_idt, the width of common electrodes b, the gap between each electrode a, the number of the EWC electrodes at the left N_L, the number of the EWC electrodes at the right N_R, the width of EWC electrode at the left w_EWC_L, and the width of EWC electrode at the right w_EWC_R. (**b**) The structure of the phase shifter used by the EWC IDTs. It contains the parameters of the EWC IDTs, the period of the reflector λ_ref, the number of electrodes of the reflector n_ref, the width of the EWC electrode of the reflector w_ref_EWC, and the number of the EWC electrodes of the reflector N_ref_EWC.

Figure 6a shows the structure of the phase-weighted IDT. It is a combination of common interdigital electrodes and the EWC interdigital electrodes. The EWC interdigital electrodes are located on the both sides of the common IDT. The width of the EWC interdigital electrode decreases progressively or increases progressively to control the phase.

(5) $$w_{1}\_EWC\_L=\frac{1}{2}\eta \times \lambda \_idt+k_{L}$$

(6) $$w_{1}\_EWC\_R=\frac{1}{2}\eta \times \lambda \_idt+k_{R}$$

(7) $$w_{i}\_EWC\_L=w_{1}\_EWC\_L+k_{L}^{\prime }\left(i-1\right)\;i\in \left[2,N\right]$$

(8) $$w_{i}\_EWC\_R=w_{1}\_EWC\_R+k_{R}^{\prime }\left(i-1\right)i\in \left[2,N\right]$$

(9) $$\eta =\frac{b}{a+b}$$

where η is the metallization rate, $k_{L}$ and $k_{R}$ are the additional quantities to decide the first width of the EWC electrode on the both sides, and $k_{L}^{\prime }$ and $k_{R}^{\prime }$ are the coefficients to decide the width of EWC electrodes. If $k^{\prime }$ is positive, the width increases progressively; otherwise, the width decreases progressively.

Figure 6b shows the structure of phase shifter. This phase shifter is a special DMS which contains EWC IDTs and reflectors. Two reflectors are symmetrical except for the EWC electrodes. Near the side of EWC IDTs, the electrodes of the reflectors are EWC electrodes. The width of the EWC electrodes are determined as follows:

(10) $$w_{i_{re}f_{EWC}}=\frac{1}{2}\eta \times \lambda_{ref}+k^{\prime \prime }i\;\;\;\;\;\;i\in \left[1,N\_ref\right]$$

where $k^{\prime \prime }$ is the coefficients to decide the width of the EWC electrodes. Controlling the EWC IDTs can affect the internal reflection. With the change of the internal reflector, the phase of the signal can also change.

**Figure 7:**
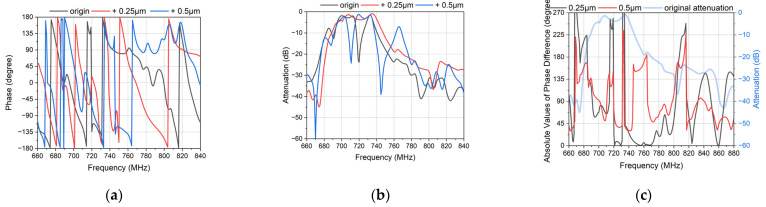
The responses of the phase shifter with different widths of the EWC electrodes. (**a**) The phase of the different widths. The width of red one is 0.25 μm larger than the width of the black one, and the width of blue one is 0.5 μm larger than the width of the black one. (**b**) The attenuation of different widths. The width of the red one is 0.25 μm larger than the width of black one, and the width of the blue one is 0.5 μm larger than the width of black one. (**c**) The absolute values of the phase difference of the different widths. The width difference of 0.25 μm is the black one and red one in (**a**). The width difference of 0.5 μm is the black one and blue one in (**a**).

Figure 7 shows the response of DMS with different widths of the EWC electrodes. Figure 7a,b show that the change of the phase is sensitive when altering the width of the EWC electrodes. Moreover, it does not affect the attenuation much which is good for the attenuators. In order to observe the phase more clearly, Figure 7c shows the absolute values of the phase difference with different widths. Among the flat passband, the phase changes more sensitively. The bandwidth of the phase is similar to the bandwidth of the passband. In the bandwidth, the phase difference can reach 180 degrees while the phase difference is usually less than 90 degrees when it is out of the bandwidth. Although the change of the phase is irregular, the EWC electrodes can ensure enough flexibility. The isolation can be improved only if the phase shifter satisfies the reverse phase. The phase can be reversed among the bandwidth by controlling the width of the EWC electrodes reasonably. 

### 3.3. Structure of Attenuator

**Figure 8:**
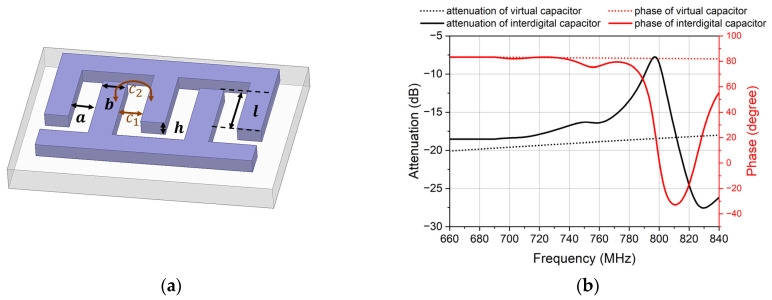
The paraments of the attenuator. (**a**) The structure of the interdigital capacitor. $C_{1}$ is the capacitance between the electrodes, and $C_{2}$ is the capacitance generated by the edge of electrodes. (**b**) The attenuation and phase of the virtual capacitor and interdigital capacitor.

The interdigital capacitor and resonator are special applications of the interdigital electrode; it is possible to manufacture interdigital capacitor by the same process and material of the resonator. This provides a possibility that the interdigital capacitor can be used as an attenuator. Figure 8a shows the structure of the interdigital capacitor. It contains the number of interdigital electrodes $N_{c}$, the gap between each interdigital electrodes a, the width of interdigital electrodes b, the aperture of interdigital electrodes l, and the thickness of interdigital electrodes h.

By controlling the capacitance, the interdigital capacitor can be designed to control the attenuation of the feedback signal to be the same as the attenuation of the leakage signal [25]. Figure 8b shows the response of the interdigital capacitor and the virtual capacitor. The number of the interdigital electrodes is 15 and the aperture is 15 μm. The capacitance of the virtual capacitor is 0.24 pF. Their attenuation and phase are similar when the interdigital capacitor is away from its resonant frequency. The smooth changes of the attenuation and phase mean the interdigital capacitor can be used as an attenuator.

The interdigital capacitor can be seen as a combination of many parallel plate capacitors, and the total capacitance is the sum of the capacitance of each parallel plate capacitor. According to the calculation formula of parallel plate capacitor, the capacitance of the interdigital capacitor is as follows:

(11) $$\;C_{0}=C_{1}+C_{2}\approx \frac{\varepsilon S}{4\pi kd}=\frac{\varepsilon lh}{4\pi ka}$$

where $C_{0}$ is the capacitance of a pair of interdigital electrodes, $C_{1}$ is the capacitance of the parallel plate capacitor, and $C_{2}$ is the capacitance of the other directions. Because $C_{2}$ is so little that it can be ignored [26], the total capacitance is as follows:

(12) $$C=C_{0}\left(N_{c}-1\right)=\frac{\varepsilon lh}{4\pi ka}\left(N_{c}-1\right)$$

where ε is the permittivity of air, k is electrostatic force constant, and $N_{c}$ is the number of the interdigital electrodes. Because the interdigital capacitor and the duplexer use the same technology, h is definite. The attenuation can be controlled by $N_{c}$, a and l. 

### 3.4. Design

**Figure 9:**
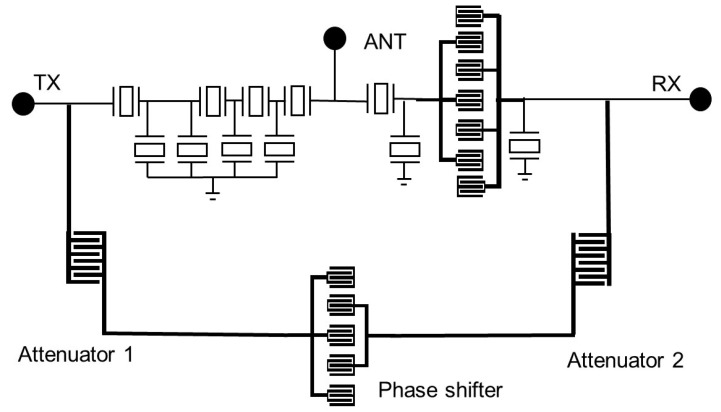
The structure of phase canceling circuit.

After the structures of the phase shifter and attenuator are determined, a phase canceling circuit can be designed to improve the isolation. A phase shifter can be designed with the DMS to reach a sufficient phase difference. Considering the size of the chip, the fifth-order DMS is the best choice. Attenuators can be designed with interdigital capacitors to reach the equal attenuation. Figure 9 shows the structure of the phase canceling circuit.

**Figure 10:**
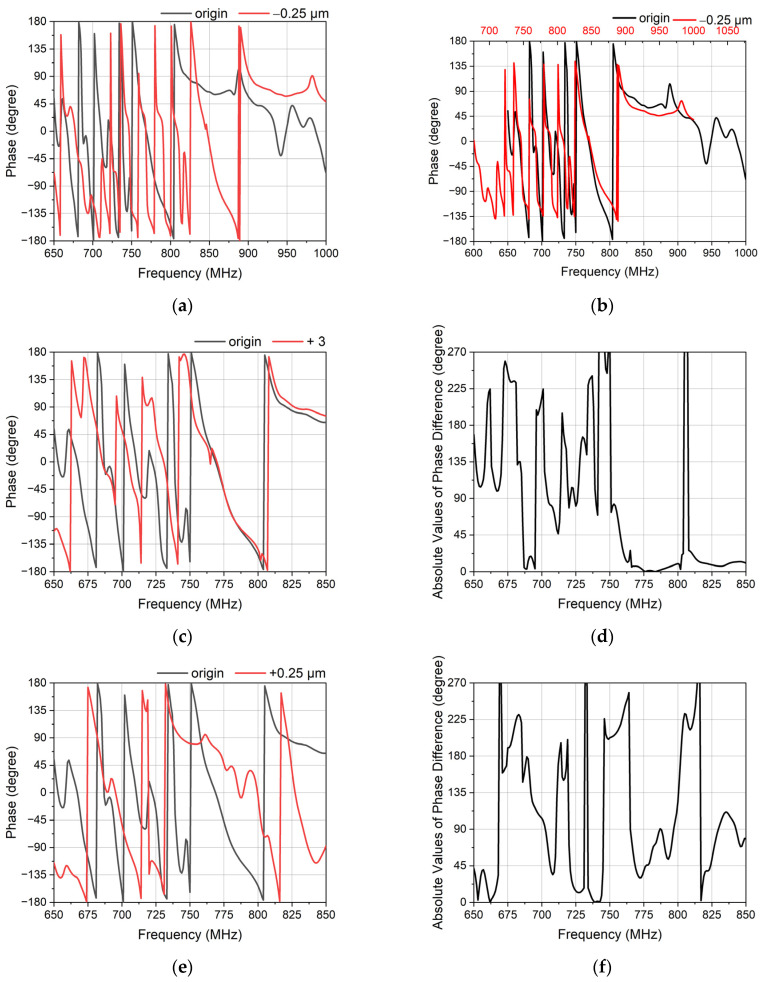
The response of the phase shifter with different parameters. (**a**) The phase of different λ. The λ of the red one is 0.25 μm less than the black one. (**b**) The attenuation of different λ. (**c**) The phase of different numbers of electrodes. The number of electrodes of the red one is 3 larger than the black one. (**d**) The absolute values of the phase difference of the different number of electrodes. (**e**) The phase of the different widths of the EWC electrodes. The widths of the EWC electrodes of the red one is 0.25 μm larger than the black one. (**f**) The absolute values of the phase difference of different widths of the EWC electrodes.

To determine the parameters, the effects of the phase shifter and attenuators were analyzed. Figure 10 shows the effects of the different paraments on the performance of the phase. Changing the λ_idt of the common IDTs changes the phase, as seen in Figure 10a. This change is affected by the frequency drift. As shown in Figure 10b, the frequency shifts about 90 MHz. The λ_idt of the common IDTs plays an important role in controlling the bandwidth rather than changing the phase. A suitable bandwidth can provide a more sensitive phase difference. Figure 10c,d show that the phase can be affected by the number of common electrodes. It can basically reach the phase difference of 90 degrees within the bandwidth. The phase difference of 180 degrees accounts for 18.5% between 650 MHz and 850 MHz. Figure 10e,f show that EWC electrodes can control the phase more easily. The phase difference of 180 degrees accounts for 25.5% between 650 MHz and 850 MHz. Both the range and the phase difference become bigger than those presented in Figure 10d. The reverse phase can be easily achieved by reasonably controlling the number of common electrodes and the width of the EWC electrodes.

**Figure 11:**
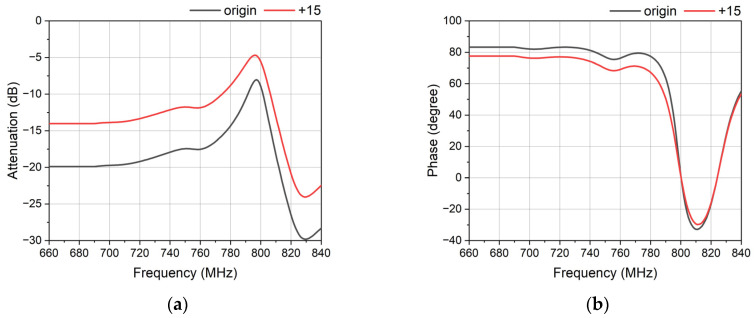
The response of the interdigital capacitor with different electrodes. The number of the electrodes of the black one is 15, and the number of electrodes of the red one is 30. (**a**) The attenuation of the different electrodes. (**b**) The phase of the different electrodes.

The equal attenuation is controlled by the interdigital capacitors. By controlling the size of the capacitors like the number of electrodes, the capacitance of the capacitor changes, which can alter the attenuation. Figure 11a shows the attenuation of the different number of electrodes. Their difference in number is 15, and the difference of their attenuation is 5.9 dB. This means that the attenuation decreases by 5.9 dB when the number of electrodes increases by 15. The change of number has a slight influence on the phase, as shown in Figure 11b. This is good for the phase shifter in avoiding the change of the phase. 

According to the principle of the phase canceling circuit, the isolation can be improved if the branch creates an equal extent and reverses the phase signal. The λ_idt of the phase shifter mainly controls the bandwidth to ensure a sensitive phase change. The λ_idt can use the λ of the DMS of the duplexer to obtain an approximate bandwidth. In order to limit the size of phase shifter, the aperture is fixed at 30 μm. n_idt should range from 5 to 30. N should range from 0 to 4. n_ref should range from 3 to 8. λ_ref should use the same value as that of the DMS of duplexer. n_ref and N_ref should range from 0 to 5. The number of common electrodes and EWC electrodes controls the phase to achieve the reverse phase. Because the influence of their parameters on the phase are complex, their values can be achieved using the trial-and-error method. Two capacitors change their number of electrodes to obtain equal attenuation. Considering that the substrate is piezoelectric material, the interdigital capacitor is influenced by the resonant frequency. The λ of the interdigital capacitors should be far away from the λ of the phase shifter. Meanwhile, the aperture of the two interdigital capacitors should be below 30 μm so that the size of the chip will not expand. Table 1 shows the optimizations of the phase shifter and attenuators.

**Figure 12:**
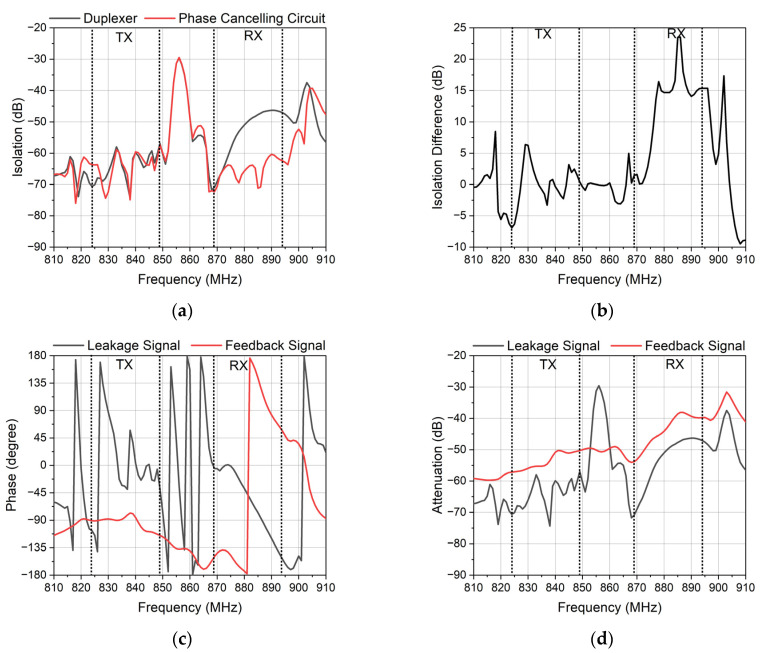

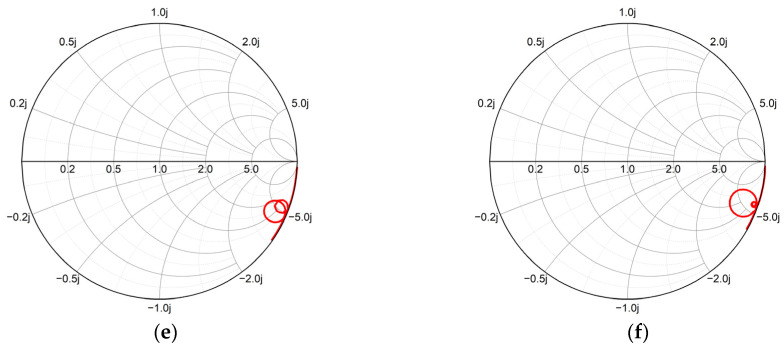
The simulation characteristics of the phase canceling circuit. (**a**) The isolation of the duplexer and the phase canceling circuit. (**b**) The improvement of isolation. (**c**) The phase of the feedback signal and leakage signal. (**d**) The attenuation of the feedback signal and leakage signal. (**e**) The Smith chart of the branch at the RX band. (**f**) The Smith chart of the branch at the TX band.

Figure 12 shows the simulation characteristics of the optimization. Figure 12a shows that the isolation is improved. The isolation is improved over 10 dB in the RX band while the isolation is unchanged in the TX band. Figure 12b shows the specific data. The isolation in the RX band is mainly improved by 15 dB. The average value is 12 dB, but the isolation is improved slightly in the TX band. Figure 12c,d show the phase and attenuation of the feedback signal and leakage signal. Compared with the phase of the leakage signal, the phase of the feedback signal meets the requirement of the reverse phase in the RX band, and the attenuation of the feedback signal is similar to the attenuation of the leakage signal. This branch meets the requirements of canceling out the leakage signal generally which leads to the improvement of isolation in order to ensure that the branch does not affect the duplexer. Figure 12e,f shows the impedance of the branch in the TX and RX bands. From the Smith chart, we can know that the import impedance and outport impedance of the branch is close to the open circuit. This can ensure that the branch will not have a big influence on the duplexer.

## 4. Results and Discussion

**Figure 13:**
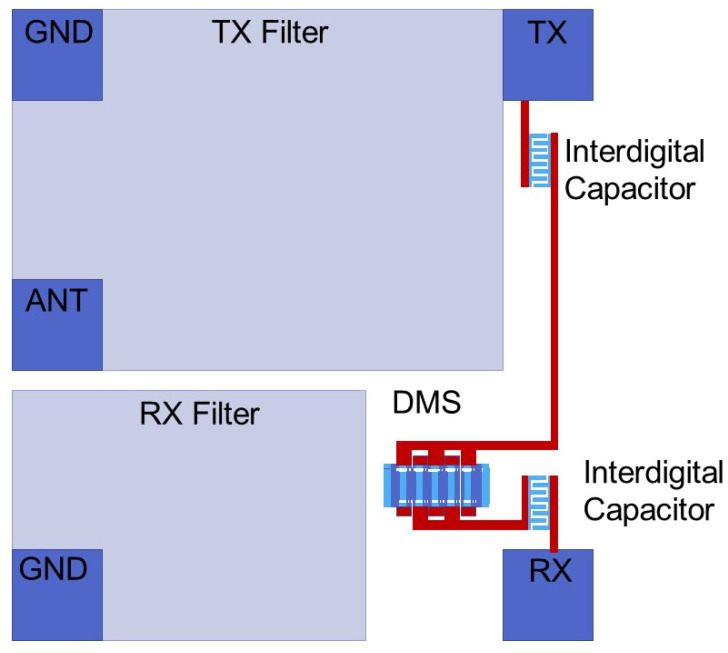
The layout of the phase canceling circuit.

In order to verify the feasibility of the phase canceling circuit, a practical circuit was made. Figure 13 shows the layout of the phase canceling circuit. In order to ensure the size of the chip, the branch surrounds the edge of the duplexer. Because the area of RX is less than the area of TX, the phase shifter is set near the RX. The attenuators are placed near the TX port and the RX port to reduce the capacitive coupling between the interdigital capacitors and the duplexer. The direction of the two small interdigital capacitors is perpendicular to the resonators of the duplexer. Finally, the sum of the width of the interdigital capacitors and the width of the track should be less than the width of the pad, which fixes the size of the chip. 

**Figure 14:**
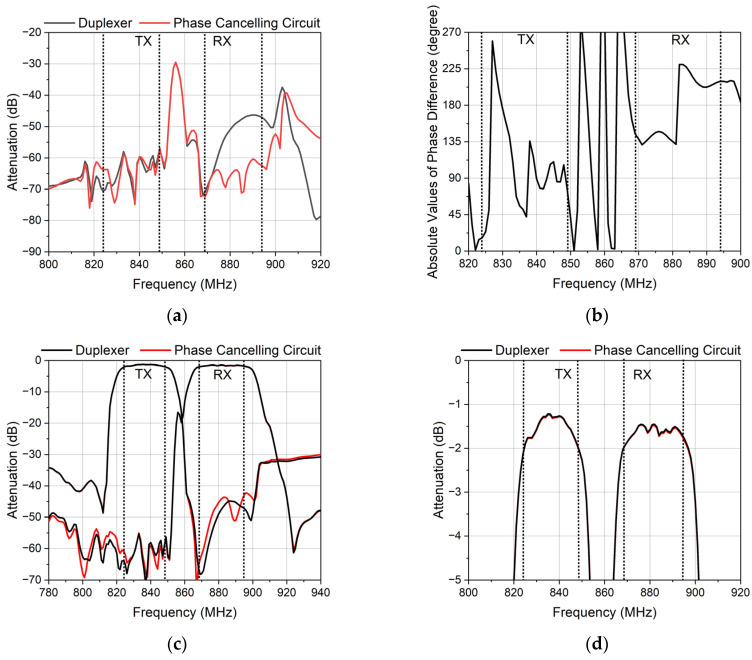
The measured characteristics of the duplexer and the phase canceling circuit. (**a**) The isolation of the duplexer and the phase canceling circuit. (**b**) The absolute values of the phase difference of the feedback signal and the leakage signal. (**c**) The attenuation of the duplexer and the phase canceling circuit. (**d**) The insertion loss of the duplexer and the phase canceling circuit.

Figure 14 shows the measured results of the phase canceling circuit and the duplexer. Figure 14a shows the isolation of the TX and the RX. The improvement of the isolation did not cover the TX and RX band wholly. In the TX band, the isolation was ameliorated partly. The isolation of the duplexer was 58 dB in the TX band and 46 dB in the RX band. The isolation of the phase canceling circuit was suppressed from 46 dB to 60 dB in the RX band. However, the isolation in the TX band was immune to the phase canceling circuit. The improved value was around 0.1 dB. 

Figure 14b shows the phase difference of the feedback signal and the leakage signal. From 828 MHz to 833 MHz, the phase difference reached 135 degrees which lead to slight improvement. From 833 MHz to 849 MHz, the phase difference reached 90 degrees, and the isolation was not improved. From 869 MHz to 894 MHz, the phase difference reached 135 degrees, and the isolation was improved obviously. Notably, the isolation improved 15 dB when the phase difference reached 180 degrees from 882 MHz to 894 MHz. It seems that the isolation can be improved if the phase difference reaches at least 135 degrees.

Figure 14c shows the attenuation of the TX and the RX. The suppression of the RX at the TX band was basically consistent to that of the duplexer, while the suppression of the TX at the RX band was different from that of the duplexer. At 885 MHz, the phase canceling circuit had an extra zero point, and it had a slight impact on the out-of-band suppression. This means the branch has an influence on the signal between the TX port and the ANT port, and the signal between the RX port and the ANT port was not affected by the branch.

Figure 14d shows the insertion loss. The insertion loss did not change. This means the phase canceling circuit is successful because the important targets of the duplexer are not affected while the isolation is improved at the same time.

One possible reason for the disappointing isolation in the TX band is the phase shifter. The phase difference between the feedback signal and the leakage signal in the RX band reached approximately 180 degrees. This is important to ensure the duplexer has a high isolation. Unexpectedly, the phase in the TX band changed little. This may be due to the poor isolation in the TX band. The bandwidth of the fifth-order DMS did not achieve a sufficient range to cover the TX band and the RX band. 

Another reason is the interdigital capacitor which was used as the attenuation. The track between the interdigital capacitor and the TX port works as an inductor. This inductor is connected in a series with the interdigital capacitor which causes an unexpected resonance in the TX band. 

## 5. Conclusions

In this paper, a phase canceling circuit was discussed to improve the isolation of the duplexer, and the validity of the structure was verified through experimental verification. The improvement of 12 dB in the RX band isolation was achieved without changing the passband insertion loss, and the return loss was also maintained at a good level. Moreover, the area of the chip was not increased. The phase canceling circuit is expected to be used at any duplexing bands to achieve higher isolation.

## Figures and Tables

**Table 1:** The parameters of the branch.

Parameters		Values				
		1	2	3	4	5
DMS_IDT	*n_idt*	8	15	28	20	8
	*width*	1.16	1.16	1.224	1.172	1.172
	*N_L*	3	3	3	3	2
	*w_EWC_L*	1.279	1.279	1.304	1.038	1.038
	*N_R*	0	0	4	2	4
	*w_EWC_R*	0	0	1.317	1.304	1.304
DMS_REF	*n_ref*	5	5			
	*w_ref*	1.182	1.182			
	*N_ref*	3	1			
	*w_ref_EWC*	1.172	1.113			
Capacitor	*N_c_*	29	29			
	*width*	1	1			
	*l*	15	15			

## Data Availability

Not applicable.
